# Book Notices

**Published:** 1881-02-20

**Authors:** 


### *BOOK NOTICES.*

HAND BOOK OF URINARY ANALYSIS: CHEMICAL AND MICROSCOPICAL. For the use of Physicians, Medical Students and Clinical Assistants. By Frank M. Deems, M. D., Laboratory in the Medical Department of the University of New York: Member of the N. Y. County Medical Society; Member of the New York Microscopical Society, etc., 12 mo.. Limp Cloth, 25 cents. New York: Indusdustral Publication Co.

"This Manual presents a plan for the systematic examination of liquid urine, urinary deposits, and calculi. It is compiled with the intention of supplying a concise guide, which, from its small compass and tabulated arrangement, renders it admirably adapted for use, both as a bedside reference book and a work-table companion. The author is well known as one who has had for several years a very extended experience as a teacher of this important branch of physical diagnosis, and he has compiled a manual which will serve to lessen the difficulties in the way of the beginner, and save valuable time to the busy practitioner. The arrangement of matter, and the small though clear type in which it is printed, has enabled the author to compress a great deal into a very small compass, so that, while serving all purposes of an analytical table, it is really a good deal more, although it is not, of course, to be supposed that this brochure can take the place of larger books."

HOW PERSONS AFFLICTED WITH BRIGHT'S DISEASE OUGHT TO LIVE. By Joseph F. Edwards, Philadllphia. Presley & Blakiston, 1012 Walnut St., 1881.

A little work of 87 pages, containing some very valuable thoughts and suggestions.

THE BACTERIA. By Dr. Antoine Magnin, Licenciate of Natural Sciences; Chief of the Practical in Natural History to the Faculty of Medicine of Lyons; Laureate of the Faculty of Medicine of Paris (silver medal, 1876); General Secretary of the Botanical Society of Lyons; Member of the Botanical Society of France, etc. Translated by George M. Sternburg, M. D., Surgeon United States Army. Boston; Little, Brown & Co., 1880.

This is a very timely work by Dr. Sternburg—the translation of the admirable treatise of Dr. Magnin, in which we have perhaps the best resume of what is known in the department of Micro-Organisms anywhere to be found. It is timely because of the great interest now taken in the subject, as a result of the action of our Government in the appointment of a National Board of Health, whose investigations it is hoped will result in new developments and new light upon a subject very much neglected and little understood by the profession in this country.

